# Randomized Clinical Trial: Effects of β-Hydroxy-β-Methylbutyrate (HMB)-Enriched vs. HMB-Free Oral Nutritional Supplementation in Malnourished Cirrhotic Patients

**DOI:** 10.3390/nu14112344

**Published:** 2022-06-03

**Authors:** Silvia Espina, Alejandro Sanz-Paris, Yolanda Gonzalez-Irazabal, Patricia Pérez-Matute, Fernando Andrade, Beatriz Garcia-Rodriguez, Christian Carpéné, Alexia Zakaroff, Vanesa Bernal-Monterde, Javier Fuentes-Olmo, Jose M. Arbones-Mainar

**Affiliations:** 1 Gastroenterology Department, Miguel Servet University Hospital, 50009 Zaragoza, Spain; silespina@gmail.com (S.E.); vbernalm@gmail.com (V.B.-M.); fuentesolmo@gmail.com (J.F.-O.); 2 Instituto de Investigación Sanitaria (IIS) Aragon, 50009 Zaragoza, Spain; sanzparisalejandro@gmail.com (A.S.-P.); yolgonira@gmail.com (Y.G.-I.); 3 Nutrition Department, Miguel Servet University Hospital, 50009 Zaragoza, Spain; 4 Clinical Biochemistry Department, Miguel Servet University Hospital, 50009 Zaragoza, Spain; bea_garcia_rodriguez@hotmail.com; 5 Infectious Diseases, Microbiota and Metabolism Unit, Center for Biomedical Research of La Rioja (CIBIR), 26006 Logroño, Spain; cpperez@riojasalud.es; 6 Metabolomics Platform, Biocruces Bizkaia Health Research Institute, 48903 Barakaldo, Spain; fernando.andradelodeiro@osakidetza.eus; 7 Institute of Metabolic and Cardiovascular Diseases, INSERM, UMR1297, Team Dinamix, 31432 Toulouse, France; christian.carpene@inserm.fr (C.C.); alexia.zakaroff@inserm.fr (A.Z.); 8 Institute of Health and Medical Research, University Paul Sabatier, 31062 Toulouse, France; 9 Translational Research Unit, Instituto Aragonés de Ciencias de la Salud (IACS), Miguel Servet University Hospital, 50009 Zaragoza, Spain; 10 Centro de Investigación Biomédica en Red Fisiopatología Obesidad y Nutrición (CIBERObn), Instituto Salud Carlos III, 28029 Madrid, Spain

**Keywords:** cirrhosis, liver function test, nutrition, HMB, supplement

## Abstract

β-Hydroxy-β-methylbutyrate (HMB) supplementation increases muscle and strength mass in some muscle-wasting disorders. Malnutrition and sarcopenia are often present in liver cirrhosis. We aimed to investigate the effects of oral HMB supplementation on changes in body composition and liver status in patients with cirrhosis and malnutrition. In a randomized, controlled, double-blind trial, 43 individuals were randomized to receive twice a day and for 12 weeks an oral nutritional supplement (ONS) enriched with 1.5 g of calcium HMB per bottle or another supplement with similar composition devoid of HMB. Inclusion criteria were liver cirrhosis with at least one previous decompensation and clinical malnutrition. Liver function, plasma biochemistry analyses, and physical condition assessment were carried out at baseline, then after six and 12 weeks of supplementation. A total of 34 patients completed the clinical trial. An improvement in liver function and an increase in fat mass index were observed in both groups. None of the two ONS changed the fat-free mass. However, we observed an upward trend in handgrip strength and a downward trend in minimal hepatic encephalopathy in the HMB group. At the end of the trial and regardless of the supplement administered, fat mass content increased with no change in fat-free mass, while liver function scores and nutritional analytic markers also improved.

## 1. Introduction

Evidence shows that malnutrition in cirrhosis is associated with worse quality of life, a greater number of complications, and increased mortality [1]. The prevalence of malnutrition is related to the clinical stage of the injured liver; it ranges from ~20% in patients with compensated cirrhosis to more than 50% in those with decompensated liver failure [2]. The physiopathology of malnutrition in cirrhosis is characterized by a state of accelerated starvation with an increase of fatty acid oxidation and reduced utilization of glucose as a source of energy [3]. This metabolic switch is accompanied by decreased protein synthesis and increased gluconeogenesis, with skeletal muscle amino acids being the main glucogenic substrate through proteolysis [4]. Cirrhosis-associated hyperammonemia increases the expression of myostatin, which further inhibits the protein synthesis and activates muscle autophagy to provide nutrients during the state of starvation [5,6,7]. All these processes converge into a loss of muscle mass, suggesting that sarcopenia is the primary nutritional consequence of malnutrition in cirrhosis [1]. In this regard, sarcopenia has been shown to be a predictor of mortality regardless of MELD (model for end stage liver disease) [8], and it’s also independently associated with increased risk of hepatic encephalopathy [9].

The daily protein intake in malnourished cirrhotic patients or with sarcopenia should be 1.5 g/kg/day [10]. However, it is difficult to ensure that malnourished individuals with liver cirrhosis achieve adequate energy and protein intake according to nutritional recommendations, so in this context, oral nutritional supplementation (ONS) may be chosen [11]. Several studies showed that branched-chain amino acid (BCAA) supplementation is effective in downregulating protein catabolism in cirrhosis [12,13]. However, a recent Cochrane review found a positive effect of BCAA supplementation on the symptoms of encephalopathy, but no benefits on mortality, quality of life, or nutritional parameters [14]. β-Hydroxy-β-methylbutyrate (HMB) is a naturally occurring and metabolically active derivative of the BCAA leucine, and its beneficial effects on human skeletal muscle were first described by Nissen more than 20 years ago [15]. The HMB stimulates protein synthesis via the mTOR system and growth hormone/IGF-1 axis, as well as decreases protein degradation pathways via the ubiquitin proteasome and the autophagy-lysosome systems [16,17]. Mounting evidence supports that supplementation with HMB can increase muscle mass and strength and reduce muscle damage during resistance exercises [15,18,19,20,21], as well as prevent muscle loss in the elderly [22,23]. However, there are few clinical reports of the effects of supplements enriched with HMB on other muscle-wasting diseases [17]. Recently, a pilot clinical trial with HMB in compensated liver cirrhosis has shown an increase in muscle mass measured by ultrasound and muscle function without modifying the BIA or muscle strength [24].

Considering that HMB may be effective in disorders characterized by increased proteolysis, such as cachexia associated with AIDS [25,26] or cancer [27,28], we hypothesized that HMB supplementation would improve fat-free mass and muscle function in malnourished patients with decompensated cirrhosis. To test this hypothesis, we implemented a double-blind controlled trial to investigate the effects of an HMB-enriched ONS on changes in body composition and liver status in patients with decompensated liver cirrhosis and clinical malnutrition. Another treatment with an ONS devoid of HMB and with similar macronutrient composition was used as control.

## 2. Materials and Methods

### 2.1. Study Design and Population

This study is a double-blind, parallel group, randomized controlled trial conducted in the University Hospital Miguel Servet (Zaragoza, Spain) that enrolled patients with liver cirrhosis of any etiology with previous clinical decompensation in the last 2 years and clinical malnutrition class B or C screened by Subjective Global Assessment (SGA) [29]. Exclusion criteria were being <18 years old or having diabetes mellitus, corticosteroid treatment, hepatocellular carcinoma, uncontrolled infection, or orthotopic liver transplantation (OLT). Patients with recent decompensation (<3 months) due to overt hepatic encephalopathy or variceal upper gastrointestinal bleeding were also excluded as a precaution due to clinical severity.

This trial was conducted in compliance with the Declaration of Helsinki and was approved by the local ethics committee (CEIC-A, ref. PI17/0258). All study participants provided written informed consent before participating in the trial. The study was registered at www.clinicaltrials.gov (NCT03285217) and reported according to CONSORT recommendations [30].

Participants were randomized with a 1: 1 ratio to receive twice a day and for 12 weeks oral supplementation either 220 mL of Ensure^®^ Plus Advance (HMB group; 1.5 kcal/mL, 24.3% protein, 28.8% fat, and 1.5 g of calcium HMB per service) or 220 mL of Ensure^®^ Plus High Protein (HP group; 1.25 kcal/mL, 25.3% protein, 23.8% fat), both provided by Abbott Laboratories (Madrid, Spain). BCAA supplementation in both ONS was carried out at a 2:1:1 ratio (Leu:Ile:Val) according to the recommendations in liver cirrhosis [31]. Supplementary Table S1 shows the nutritional content of each ONS. Permuted-block randomization with random block size was performed using the package blockrand of R, with participants randomly assigned into one of two study groups. Allocations were independently placed in sealed numbered envelopes and were opened sequentially by a single researcher (J.M.A.-M.) after participants provided informed consent. Clinicians and laboratory staff assessing the patients were blinded to the group allocation.

### 2.2. Outcome Measures

A clinical, laboratory (venous sampling) and anthropometric assessment was carried out at baseline, then after 6 and 12 weeks of supplementation at the clinical site. Primary outcome measures included changes in body composition, in particular fat and fat-free mass, liver status measured with the Child–Pugh [32] and MELD scores [33], as well as liver transaminase enzymes: gamma-glutamyl transpeptidase (GGT), aspartate transaminase (AST), and alanine transaminase (ALT). Other pre-specified outcomes of interest were a complete hepatic evaluation of decompensation and complication events, medications, and MHE (minimal hepatic encephalopathy) evaluation by PHES (Psychometric Hepatic Encephalopathy Score) [34].

The physical assessment was performed in a fasting state and included anthropometric evaluations and a single-frequency hand-to-foot bioelectrical impedance analysis (BIA) employing the BIA 101 instrument (Akern, Pontassieve, Italy). Muscle strength evaluation was carried out using a hydraulic dynamometer in the dominant hand (handgrip). In patients with refractory ascites, BIA was performed after large-volume paracentesis. Anthropometry included body weight, biceps (BSF) and triceps (TSF) skinfold, mid-upper arm circumference (MUAC), and calf circumference. Mid-arm muscle circumference (MAMC) was calculated from MUAC and TSF. Muscle mass and adiposity indices were estimated by BIA using the Bodygram Plus^®^ program, including fat mass index (FMI) and fat-free mass index (FFMI). Other measures of body composition by BIA were also included: body mass index (BMI), fat mass (FM), percentage of fat mass (%FM), fat-free mass (FFM), percentage of fat-free mass (%FFM), total body water (TBW), and body cell mass (BCM).

Laboratory work was performed at the Clinical Biochemistry Department. Analyses included parameters of nutrition (plasma proteins, albumin, prealbumin, folic acid, and vitamin B12), hematimetric indices (hemoglobin, INR and blood cell count), cardiovascular risk markers (triglycerides, total cholesterol, HDL-cholesterol, LDL-cholesterol, apolipoprotein A (APOA), apolipoprotein B (APOB), and lipoprotein (a), bone turnover markers (vitamin D and osteocalcin), as well as other metabolic and liver functionality markers (ferritin, transferrin, C reactive protein, urea, creatinine, bilirubin, alkaline phosphatase, and ammonia).

HMB determination employed a gas chromatography-mass spectrometry methodology. Briefly, 25 μL of internal standard solution (tropic acid 6 mM in MeOH) was added to 975 μL of plasma. The solution was extracted twice with 2 mL of ethyl acetate. The organic phase was then transferred into a second tube and evaporated to dryness under nitrogen at 50 °C. The evaporation residue was mixed with 50 μL of pyridine and 50 μL of BSTFA (N,O-Bis(trimethylsilyl)trifluoroacetamide) for derivatization. This mix was incubated 30 min at 60 °C and finally injected into the chromatograph.

### 2.3. Statistical Analyses

Statistical analysis was carried out in R 3.4.0. and the appropriate packages according to the predefined statistical analysis plan. Results are present as means and SDs for normal variables or medians and interquartile ranges (IQRs) for non-normal data. For the longitudinal analysis, we used an intent-to-treat (ITT) approach which included all participants randomized, regardless of whether they finished the full study protocol. The data were modeled using linear mixed-effects models (LMM) for repeated measures using the *lme* function of the n*lme* package to take into consideration (1) the repeated assessment of each variable and (2) the existence of missing values. Missing values in outcome variables were not imputed. LMM models produced different *p*-values that captured the variation over time of each variable for the entire cohort (p*long*) and treatment-specific longitudinal changes, that is, the interaction between longitudinal changes and treatment (plong*treatment). The power for detecting treatment-specific longitudinal changes in the muscle mass index in a sample of 21 participants was over 80% according to power simulations run using the *longpower* package for LMMs.

## 3. Results

### 3.1. Baseline Characteristics

**Figure 1:**
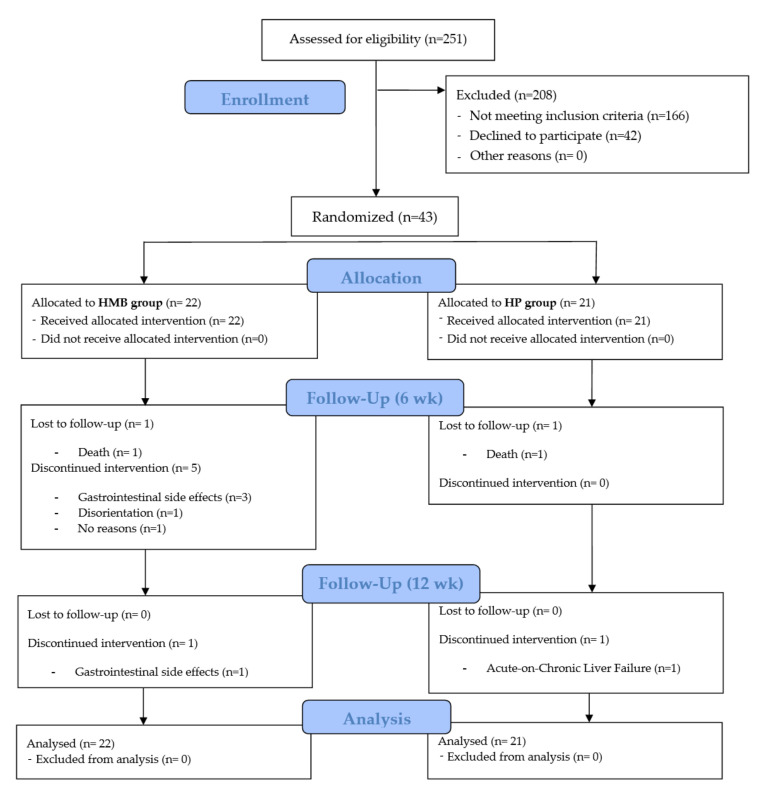
Study flow diagram.

Between July 2017 and January 2018, 251 individuals were screened and 43 randomized to receive oral supplementation twice a day with either 220 mL of Ensure^®^ Plus Advance (HMB group, *n* = 22) or oral supplementation twice a day with 220 mL of Ensure^®^ Plus High Protein (HP group, *n* = 21) (Figure 1).

A total of 34 patients completed the clinical trial (68% in the HMB group and 90% in the HP group). Table 1 shows the overall distribution of baseline characteristics of the participants in the trial by treatment group assignment. There were no significant differences between treatment groups with respect to age (*p* = 0.711), sex (*p* = 1.000), or etiology of cirrhosis (*p* = 0.624).

Alcohol use was the main etiology in both groups. At the time of enrollment, the prevalence of ascites, MHE, and previous encephalopathy were similar in both groups. The global median (interquartile range) of MELD score was 12 (8.5;16.5) and of Child–Pugh score was 7 (6.0;8.5), without differences between groups (*p* = 0.835 and *p* = 0.398, respectively). For the nutritional status assessment, the SGA scale was used, finding no differences between groups (*p* = 0.355), with 72% of patients assessed as moderately malnourished (B) and the other 28% as severely malnourished (C). On all of the other variables, including body composition (BIA, anthropometry, and handgrip), diabetes control, plasma lipid and lipoprotein levels, liver function test, iron tests, serum albumin and prealbumin, and ammonium, the groups were well matched (Supplementary Tables S2 and S3). It is worth noting that, compared to our laboratory reference values, plasma levels of prealbumin, osteocalcin, and vitamin D were decreased while the levels of bilirubin, AST, GGT, and ammonia appeared elevated at the baseline with respect to the values in the general population. Likewise, we observed decreased values in the triceps skinfold in men.

### 3.2. Longitudinal Changes in Body Composition, Handgrip Strength, and Liver Status

BIA analysis showed a longitudinal increase in the BMI (p*long* = 0.002) and a ~20% increase in the fat mass at the end of the clinical trial (p*long* = 0.024) (Table 2). This translated into similar rises in the fat mass index (p*long* = 0.014) and in the percentage of fat mass (p*long* = 0.029), without differences between sexes. We also observed a longitudinal 5% decrease in the percentage of fat-free mass (p*long* = 0.029), although the fat-free mass index did not change significantly (p*long* = 0.718). There was no variation in the total body water (p*long* = 0.819) or the body cell mass (p*long* = 0.069). With regard to anthropometric measures, there were longitudinal increases in the body weight (p*long* = 0.002) and a median 0.5 cm gain in the tricipital fold (p*long* = 0.015), while a median 0.5 cm decrease occurred in the circumference of the calf (p*long* = 0.037), with no differences between sexes or treatments. Finally, we did not observe longitudinal differences in handgrip strength in either of the two treatment groups (p*long* = 0.095) (Table 2).

**Figure 2:**
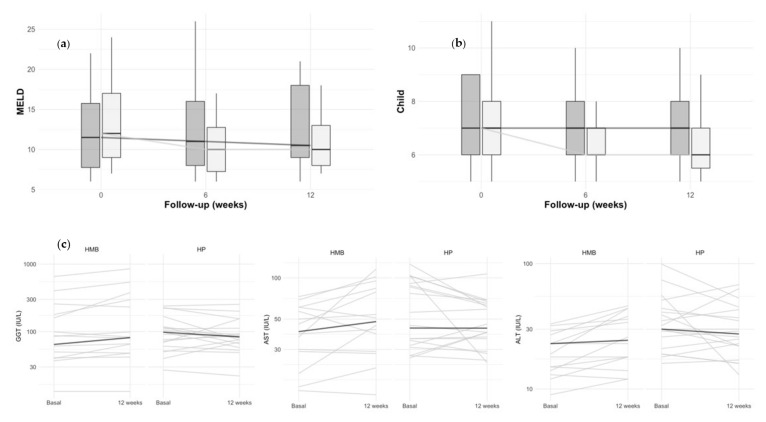
Longitudinal changes in MELD, Child-Pugh scale and in liver transaminases. Longitudinal changes in the MELD (**a**) and Child-Pugh (**b**) scales according to HMB (white) and HP (gray) treatments. (**c**) Changes in liver transaminases. Each line represents the variation of an individual patient during the trial, thick lines represent the medians of each group.

At the end of the clinical trial, a significant longitudinal decrease in MELD score was shown in both groups (p*long* = 0.02), with no differences between treatments (plong*treatment = 0.078) (Figure 2a). A similar downward trend was observed in the Child–Pugh score during treatment, although without reaching statistical significance (p*long* = 0.081) (Figure 2b). Supplementation was associated with a longitudinal increase in the plasma values of liver enzymes GGT (p*long* = 0.01) and AST (p*long* = 0.039) while no changes occurred in ALT (p*long* = 0.125). However, a treatment-dependent effect emerged as we observed increases after HMB treatment compared to baseline in GGT (25%) and AST (13%) while HP supplementation decreased GGT (−14%), AST (−7%), and ALT (−14%). Those changes were modest but significant (plong*treatment = 0.023, 0.004, and 0.032 for GGT, AST, and ALT respectively) (Figure 2c). 

### 3.3. Longitudinal Changes in Plasma Biochemistry Analyses and Nutritional Status

With regard to analytical parameters (Table 3), after oral supplementation, there was a longitudinal decrease in LDL cholesterol (p*long* = 0.002) and APO-B (p*long* < 0.001), without differences between treatments. Other lipid parameters (triglycerides, HDL cholesterol, APO-A, lipoprotein a) did not vary at the end of the study. There was a significant decrease in C-reactive protein (CRP) (p*long* = 0.044), with no differences between groups, while there were no changes in lactate dehydrogenase (LDH). The intact osteocalcin levels increased significantly (p*long* < 0.001), with no differences between treatments. Treatment with the HMB-enriched supplement was significantly associated with a longitudinal increase in the plasma levels of vitamin D (plong*treatment < 0.001, p*long* < 0.001) and HMB (plong*treatment < 0.001, p*long* < 0.001) as both compounds are part of the ONS enriched with HMB. Importantly, neither of the two treatments significantly increased plasma ammonia (Table 3). After oral supplementation for 12 weeks, a longitudinal increase in prealbumin (p*long* < 0.001), folic acid (p*long* < 0.001), and transferrin (p*long* < 0.001) values was observed, without significant differences between treatments. There were no significant changes in albumin, creatinine, vitamin B12, or total protein during the trial for either group. Neither change was observed in the hematimetric indices (leukocyte and platelet count, hemoglobin, and INR) (Table 3).

### 3.4. Adverse Events

Gastrointestinal effects were the main reason for dropout, especially in the HMB group (18.1%). No differences in clinical or biochemical characteristics were observed between compliers and dropouts (Supplementary Table S4).

During the clinical trial, there were no significant differences between the treatments in the events that led to hospital admission or severe clinical complications; ascites (*n* = 3 for HMB group vs. *n* = 4 for HP group, *p* = 1.000), hepatic encephalopathy (*n* = 3 for HMB group vs. *n* = 2 for HP group, *p* = 1.000) or digestive bleeding due to varices (*n* = 2 for HMB group vs. *n* = 1 for HP group, *p* = 0.606), infection (*n* = 2 for HMB group vs. *n* = 2 for HP group, *p* = 1.000), renal failure (*n* = 2 for HMB group vs. *n* = 2 for HP group, *p* = 1.000), acute-on-chronic liver failure (*n* = 0 for HMB group vs. *n* = 1 for HP group, *p* = 1.000), hepatocarcinoma (*n* = 0 for HMB group vs. *n* = 0 for HP group, *p* = 1.000), or death (*n* = 1 for HMB group vs. *n* = 1 for HP group, *p* = 1.000). Although not significant, at the end of the trial, the diagnosis of MHE decreased by 16.4% in the HMB group while it increased by 2.1% in the HP group.

## 4. Discussion

This clinical trial investigated the effects on malnourished decompensated cirrhotic patients of two different and commercially available ONS; one enriched with HMB and the other without HMB, both with similar macronutrient composition. At the end of the clinical trial, an improvement in liver function scores and an increase in body mass index and fat mass content were observed, while serum concentrations of LDL cholesterol and apolipoprotein B were reduced. Since the expected clinical trajectory of these patients would be towards the loss of body fat [35,36], it is likely that ONS were responsible for body composition changes. There was no significant difference between the two treatments with respect to changes in body composition, liver disease status, or in preventing muscle strength decline. However, an increase in liver GGT and AST activities was only observed in patients treated with HMB-enriched supplements.

HMB has previously been investigated in the context of cachexia, a condition characterized by weight loss and muscle similar to what is observed in malnourished cirrhotic subjects. Bear et al. recently reviewed the effects on cachexia of oral HMB alone or associated with other low-energy nutrients showing an improvement in skeletal muscle mass and muscle strength with no changes in body fat [16]. Similar results were found by Holeček M in another review [17] in which oral HMB supplementation also showed positive results on anthropometric parameters in individuals with chronic obstructive pulmonary disease, hip fracture, cancer, and AIDS while no effects were found in patients with rheumatological disease, kidney failure, or gastric bypass. Supplementation for three months with HMB in patients after surgical procedures that required prolonged hospitalization also improved nutritional and anthropometric parameters, with excellent tolerance [37]. Two recent works have reported the effects of the same HMB-enriched high-energy ONS used in our study (Ensure^®^ Plus Advance). In the first one, treatment with the HMB ONS increased muscle mass and BMI without increasing intramuscular adiposity in pre-frail older persons without active or uncontrolled conditions, when compared to a non-active placebo [38]. In the second one, the HMB-enriched supplement increased BMI, FFM, FM, BCM, and handgrip strength in patients with or at risk of malnutrition (63% being cancer patients) compared to standard ONS [39].

To date, there is no clinical trial evaluating the effect of HMB on the anthropometry of individuals with decompensated liver cirrhosis. In our study, oral supplementation for 12 weeks did not change fat-free mass in our patients with decompensated liver cirrhosis. After 12 weeks, we observed an increase, both in the HMB and in the HP group, in fat mass (*p* = 0.024), in the fat mass index (*p* = 0.014), and in the percentage of fat mass (*p* = 0.029), without differences between sexes. This contrasts with some previous reports. One clinical trial in men showed that fruit juice supplemented with HMB after orthotopic liver transplantation (OLT) increased SMI, MAMC, and handgrip strength compared to the control group (placebo with fruit juice) [40]. The same group also investigated the effect of oral HMB alone in compensated liver cirrhosis (Child–Pugh A/B) and observed an increase in muscle mass and muscle function tests without modification of BIA or handgrip strength [24]. The increase in fat mass, observed in our trial and not in the aforementioned studies might be explained, at least partially, by the high energy content of the ONS, providing approximately 550–660 additional kcal per day. This is in line with the results using Ensure^®^ Plus Advance in which an increase in BMI and/or fat mass was also observed [38,39].

Oral supplementation for 12 weeks did not change fat-free mass in our patients, both in the HMB group and in the HP group, since at the end of the study there were no changes in the fat-free mass index (*p* = 0.718). This contrasts with an increase in skeletal muscle mass observed with previous studies using HMB-enriched ONS [38,39,40]. However, supplementation with HMB in compensated liver cirrhosis also did not increase lean mass by BIA [24]. Muscle loss in cirrhosis is a complex process involving many factors and fat-free mass gain appears to be more difficult than other muscle-wasting conditions. We hypothesize that the muscle mass recovery in liver cirrhosis would require a longer supplementation time, possibly associated with physical exercise. It can be argued that BIA may falsely alter FFMI and FMI when extracellular water increases, as occurs in patients with ascites. However, the prevalence of ascites did not change in any group at the end of the clinical trial (*p* = 0.69 for HMB group vs. *p* = 0.15 for HP group)

We observed that oral supplementation also did not significantly increase muscle strength (*p* = 0.095), although we observed in the HMB group a trend to improve by 13% the handgrip strength basal level, while this increase was only of 3% in HP group. According to the bibliography, high energy HMB-enriched supplements did not change handgrip strength in either older patients [41] or in those with compensated liver cirrhosis [24]. However, an increase was demonstrated after OLT [40] and in malnourished patients [39]. Our borderline effect warrants larger studies in which statistical significance may be reached.

Regarding the effect on liver function, we observed at the end of the clinical trial in both groups a significant improvement in the MELD score (*p* = 0.02), and a trend towards an improved Child–Pugh scale was also observed (*p* = 0.081). To date, the only clinical trial to evaluate HMB in liver cirrhosis did not find significant differences in MELD or Child–Pugh score after 12 weeks of HMB supplementation [24]. As for clinical events (ascitic decompensation, gastrointestinal bleeding, hepatic encephalopathy) or complications (infection, renal failure, ACLF, hepatocarcinoma or death), there were no differences between groups. Treatment with HMB non-significantly reduced minimal hepatic encephalopathy, similar to the existing literature with BCAAs supplementation in liver cirrhosis [14,42,43]. 

Oral supplementation with HMB was also associated with increased plasma levels of vitamin D (*p* ≤ 0.001) and HMB (*p* = 0.003), both components of the Ensure Plus Advance^®^, providing further evidence of adherence to treatment. HMB supplementation demonstrated positive effects of HMB on bone density in a previous trial when compared to no supplementation [44]. We did observe an increase of osteocalcin, marker of bone absorption, along the study. A similar increase was also observed in folic acid levels. However, those effects occurred with both treatments, indicating that they were not specific to HMB but rather to energy supplementation or some other component shared by both ONS. There was also a significant increase in both groups at the end of the trial in prealbumin, which was decreased at baseline, but not in other nutritional laboratory markers that were within normal range at baseline, such as albumin, total protein, and vitamin B12. Statistical models used to test for differences were also able to capture a marginal (but significant) decrease over time in LDL-cholesterol and CRP for the entire cohort, although changes in the HP group accounted for most of this longitudinal variation. Larger trials would be required to test whether those changes may have clinical repercussions.

The most serious adverse events during the trial occurred similarly in both treatment groups and were probably due to the high vulnerability of the patients enrolled. Unexpectedly, and although in a non-hepatotoxic range (<2 × ULN), HMB supplementation increased the liver enzymes GGT (*p* = 0.023) and AST (*p* = 0.004) compared to the HP treatment. Previous studies had not described any change in the levels of liver enzymes with HMB supplementation [24], after OLT [40], or with BCAA supplementation in liver cirrhosis [42]. In this trial, the HMB group had a slightly larger proportion of alcoholic cirrhotic etiology at baseline (77% HMB group vs. 52% HP group). We posit that the increase of liver enzymes in the HMB group may be partially explained by an interaction between HMB, damage to the liver from years of excessive alcohol consumption, and the co-administration of high-energy nutrients. Consequently, more studies to investigate this interaction should be carried out.

This is the first clinical trial to evaluate the effect of HMB in patients with both decompensated liver cirrhosis and clinical malnutrition. The main limitation of this study is the high dropout rate in the HMB group (31.8%) which differed from the HP group (9.5%). After a sensitivity analysis and a comparison between compliers and dropouts (Supplementary Table S4), we considered these dropouts as missing at random (i.e., their outcomes would have been the same whether they had dropped out or not). Moreover, to reduce the possible bias in the estimate of treatment effect caused by dropout, we used an intention-to-treat approach as well as a mixed models analysis that can yield unbiased estimates of treatment effect [45]. It is worth noting that HMB in combination with other nutrients included in the ready-to-use supplement seems to influence tolerability, with a dropout rate in this group of 18.1% due to digestive symptoms (nausea, vomiting, and abdominal pain). Another study with the Ensure^®^ Plus Advance in elderly had only 9% of participants reporting gastrointestinal disorders [41]. Other reasons to drop out, such as death or liver failure did not seem to be associated to ONS treatment, but to the high vulnerability of decompensated cirrhosis itself. 

The reduced sample size of the trial could be considered as another limitation. However, it is in line with other clinical trials with HMB in compensated liver cirrhosis (*n* = 24) [24] or after OLT (*n* = 22) [40]. Although the small sample size may reduce the strength of this finding, it should be investigated whether a new formulation for HMB would be better tolerated by patients with cirrhosis. It should be also noted that the HMB group received the Ensure^®^ Plus Advance formula with a caloric content 20% higher than Ensure^®^ Plus High Protein (HP treatment). However, we do not think that this difference in composition significantly influenced the results as ONS accounted for only ~30% of the daily intake of the patients. This represents a 110 Kcal difference between ONS in a total daily intake of 1800–2000 kcal. Moreover, the increase observed in fat mass and fat mass percentage occurred in both HMB and HP groups.

## 5. Conclusions

In summary, with both ONS for 12 weeks, with or without HMB, an increase in fat mass with no benefit on fat-free mass was observed, as well as improvements in liver function scores and nutritional laboratory markers. Patients on HMB-enriched supplements trended towards improved muscle strength and reduced MHE. However, as a precaution, non-HMB supplements should preferentially be used in cirrhotic patients until other larger trials are performed to confirm or refute the alterations in liver function tests that we have observed.

## Figures and Tables

**Table 1:** Demographics and baseline characteristics.

	HMB Group (*n* = 22)	HP Group (*n* = 21)	*p*
Age (Years)	60.4 ± 8.61	61.4 ± 9.27	0.711
Sex (Men/Female)	14 (63.6%)/8 (26.4%)	13 (61.9%)/8 (38.1%)	1.000
Etiology *n* (%)			
Alcohol	17 (77.3%)	11 (52.4%)	0.624
HCV	2 (9.09%)	3 (14.3%)	
Autoimmune	2 (9.09%)	2 (9.52%)	
NAFLD	1 (4.55%)	2 (9.52%)	
HBV + NAFLD	0 (0%)	1 (4.76%)	
PBC	0 (0%)	1 (4.76%)	
Hemochromatosis	0 (0%)	1 (4.76%)	
Ascites	12 (54.5%)	9 (42.9%)	0.645
Refractory ascites	4 (18.3%)	0 (0%)	0.108
Previous encephalopathy	2 (9.09%)	3 (14.3%)	0.664
MHE (PHES) ¹	8 (36.4%)	4 (19%)	0.355
Child-Pugh			0.398
Class A	9 (40.9%)	10 (47.6%)	
Class B	11 (50%)	11 (52.3%)	
Class C	2 (9.09%)	0 (0%)	
MELD	12.7 ± 5.31	13 ± 4.7	0.835
SGA			0.355
Class B	14 (63.6%)	17 (81.0%)	
Class C	8 (36.4%)	4 (19.0%)	

Data are number (%) or mean ± SD. HCV: Hepatitis C Virus, NAFLD: Non-Alcoholic Fatty Liver Disease, HBV: Hepatitis B Virus, PBC: Primary Biliary Cholangitis, MHE: Minimal Hepatic Encephalopathy, PHES: Psychometric Hepatic Encephalopathy Score, MELD: Model for End stage Liver Disease, SGA: Subjective Global Assessment. ¹: Diagnosis of MHE by PHES.

**Table 2:** Longitudinal changes in body composition and handgrip strength.

	HMB Group			HP Group			p*long*	plong* treatment
	T0	T1	T2	T0	T1	T2		
BMI (Kg/m²)	25.4 [22.5;28.8]	27.4 [22.0;30.5]	26.9 [21.4;30.6]	26.3 [23.8;28.7]	27.1 [24.4;29.0]	25.6 [25.1;28.5]	**0.002**	0.517
FM (Kg)	13.4 [3.45;20.8]	15.8 [12.0;23.4]	16.3 [5.65;20.5]	14.2 [6.40;19.0]	17.5 [12.9;18.8]	16.4 [10.9;21.0]	**0.024**	0.651
FMI (Kg/m²)	4.85 [1.25;7.15]	6.35 [3.92;8.40]	6.20 [1.95;7.50]	5.30 [2.20;7.20]	6.05 [4.67;7.47]	6.10 [3.75;8.10]	**0.014**	0.692
%FM	18.4 [6.15;25.6]	25.5 [15.1;27.9]	21.1 [8.45;26.6]	14.2 [6.40;19.0]	17.5 [12.9;18.8]	16.4 [10.9;21.0]	**0.029**	0.684
FFM (Kg)	62.1 [48.5;64.5]	56.1 [46.6;66.3]	57.9 [46.8;64.9]	56.9 [50.3;68.1]	55.8 [51.5;62.5]	55.7 [52.3;59.6]	0.841	0.963
FFMI (Kg/m²)	21.4 [19.9;22.6]	22.2 [18.5;22.9]	19.8 [19.1;22.4]	21.7 [19.2;23.4]	20.6 [19.2;21.8]	20.3 [19.2;22.8]	0.718	0.916
%FFM	81.5 [74.5;93.8]	74.5 [72.0;84.9]	78.9 [73.3;91.6]	81.2 [73.9;91.6]	77.8 [73.2;82.9]	77.0 [71.2;86.3]	**0.029**	0.684
TBW (L)	46.5 [35.5;50.4]	44.4 [34.5;50.1]	43.2 [34.5;49.6]	41.0 [35.6;47.2]	42.0 [37.1;47.9]	41.8 [38.9;44.9]	0.819	0.194
BCM (Kg)	33.5 [27.5;37.4]	30.9 [24.3;37.8]	31.2 [23.0;38.3]	32.9 [25.1;46.1]	29.9 [23.9;40.1]	27.3 [25.5;35.5]	0.069	0.529
Body weight (Kg)	71.8 [58.5;82.9]	73.0 [57.8;88.5]	70.0 [59.5;85.5]	72.0 [64.5;79.0]	72.5 [66.9;77.8]	72.0 [66.3;76.2]	**0.002**	**0.619**
Biceps SF (mm)	6.00 [4.50;10.0]	6.00 [4.62;8.38]	7.00 [4.75;11.0]	8.00 [6.00;10.0]	8.00 [6.25;8.50]	7.00 [6.00;9.50]	0.335	0.077
Triceps SF (mm)	12.5 [8.50;14.5]	12.5 [10.2;19.0]	11.0 [9.00;18.0]	15.0 [8.50;20.0]	11.5 [8.75;18.0]	16.0 [9.50;20.5]	**0.015**	0.922
MUAC (cm)	26.0 [23.2;29.8]	27.2 [24.1;29.6]	27.5 [23.8;29.5]	28.0 [25.0;30.0]	27.5 [25.5;30.2]	28.0 [25.5;30.8]	0.111	0.956
MAMC (cm)	21.3 [19.2;25.0]	21.2 [18.0;24.6]	21.7 [20.2;24.2]	22.8 [20.7;25.0]	23.3 [20.7;25.9]	22.4 [21.2;24.9]	0.688	0.344
Calf circumference (cm)	35.0 [32.9;38.0]	34.8 [30.4;36.8]	35.0 [31.0;37.2]	36.0 [33.0;38.0]	36.0 [33.2;38.2]	35.0 [33.8;38.0]	**0.037**	0.303
Handgrip (Kg)	26.5 [23.2;34.0]	29.5 [24.8;35.2]	30.0 [24.5;33.0]	32.0 [28.0;40.0]	33.0 [26.5;38.0]	33.0 [25.5;37.0]	0.095	0.608

T0: baseline, T1: 6 wk, T2: 12 wk. Data are median [interquartile range]. BMI: body mass index, FM: fat mass, FMI: fat mass index, %FM: percentage of fat mass, FFM: fat-free mass, FFMI: fat-free mass index, %FFM: percentage of fat-free mass, TBW: total body water, BCM: body cell mass, SF: skinfold, MUAC: mid-upper arm circumference, MAMC: mid-arm muscle circumference. Linear mixed models were used to evaluate the associations of variables with multiple measurements; p*long*: variation over time of each variable for the entire cohort; plong*treatment: treatment-specific longitudinal changes. Bold *p*-values indicate significant differences.

**Table 3:** Longitudinal changes in plasma biochemistries.

	HMB Group			HP Group			p*long*	plong* treatment
	T0	T1	T2	T0	T1	T2		
Prealbumin (mg/dL)	11.6 [6.38;15.2]	12.8 [8.75;16.4]	14.5 [9.49;22.8]	9.18 [7.62;12.8]	11.2 [8.67;14.6]	10.9 [9.60;13.9]	**<0.001**	0.063
Albumin (g/dL)	3.55 [3.00;4.18]	3.70 [3.27;4.12]	3.70 [3.35;4.05]	3.50 [3.20;4.00]	3.60 [3.30;4.10]	3.50 [3.35;3.85]	0.695	0.632
Plasma proteins (g/dL)	7.20 [6.82;7.40]	7.30 [6.77;7.82]	7.40 [6.93;7.80]	7.10 [6.60;7.43]	7.10 [6.72;7.50]	7.20 [6.45;7.40]	0.551	0.358
Transferrin (mg/dL)	217 [201;259]	216 [196;281]	230 [197;316]	203 [148;252]	261 [212;303]	268 [217;308]	**<0.001**	0.757
Folic acid (ng/mL)	7.74 [6.42;9.88]	11.0 [8.39;12.0]	10.5 [6.86;12.9]	9.39 [6.69;11.6]	12.8 [8.34;14.7]	12.1 [10.3;15.5]	**<0.001**	0.816
Vitamin B12 (pg/mL)	488 [252;778]	488 [292;673]	406 [330;610]	599 [389;756]	648 [427;771]	598 [438;791]	0.942	0.432
LDL-chol (mg/dL)	100 [70.0;146]	83.5 [64.0;132]	102 [73.0;133]	100 [86.0;119]	94.0 [79.0;118]	95.0 [73.0;114]	**0.002**	0.894
HDL-chol (mg/dL)	50.0 [37.2;63.0]	49.0 [34.8;56.2]	50.0 [35.5;69.5]	53.0 [36.0;66.0]	59.0 [48.0;74.0]	56.0 [48.0;72.0]	0.114	0.619
Total chol (mg/dL)	166 [130;213]	162 [118;206]	170 [131;218]	168 [139;208]	170 [152;200]	171 [137;198]	0.078	0.687
Triglicerides (mg/dL)	68.0 [51.2;93.2]	71.0 [55.5;93.2]	80.0 [59.0;87.0]	68.0 [54.0;106]	66.0 [59.0;86.0]	68.0 [64.0;98.0]	0.858	0.679
APO A1 (mg/dL)	142 [108;168]	128 [103;174]	119 [107;171]	148 [117;170]	152 [134;185]	142 [129;177]	0.604	0.381
APO B (mg/dL)	72.3 [51.3;112]	64.2 [46.1;104]	70.9 [46.5;95.4]	70.3 [63.8;86.1]	63.8 [52.2;77.1]	65.3 [55.8;71.8]	**<0.001**	0.519
Lipoprotein a (mg/dL)	9.75 [3.21;17.1]	13.0 [3.23;23.6]	9.75 [4.22;20.8]	3.40 [2.06;7.79]	5.17 [4.13;9.93]	4.60 [3.40;10.2]	0.303	0.79
Bilirubin (mg/dL)	1.69 [1.05;2.46]	1.42 [0.84;2.31]	1.42 [0.94;2.23]	1.75 [1.31;2.57]	1.49 [0.96;2.13]	1.38 [1.00;2.08]	**0.022**	0.218
Creatinine (mg/dL)	0.73 [0.58;0.91]	0.75 [0.62;0.99]	0.80 [0.68;0.92]	0.69 [0.61;0.81]	0.75 [0.61;0.90]	0.74 [0.55;0.81]	0.608	0.672
Urea (mg/dL)	29.0 [24.5;42.5]	47.0 [28.2;66.8]	45.0 [27.5;53.0]	28.0 [25.0;34.0]	38.0 [30.0;44.0]	38.0 [25.5;41.5]	**0.048**	0.097
Ammonia (µM)	56.0 [40.0;83.0]	59.0 [50.5;85.2]	67.0 [49.8;74.8]	54.0 [39.8;78.0]	62.0 [53.2;87.2]	67.0 [55.5;75.5]	0.11	0.689
ALP (U/l)	104 [93.8;140]	107 [93.0;134]	110 [88.5;176]	131 [112;158]	127 [101;171]	126 [98.5;146]	0.594	0.172
GGT (U/l)	64.5 [40.2;100]	91.5 [50.2;146]	80.0 [51.0;264]	94.5 [58.8;120]	84.0 [54.0;95.0]	81.0 [62.0;102]	**0.01**	**0.023**
AST(U/l)	40.5 [30.8;56.5]	41.0 [27.5;61.5]	45.0 [29.5;81.5]	43.0 [32.0;88.0]	45.0 [32.0;64.0]	40.0 [34.0;64.0]	**0.039**	**0.004**
ALT (U/l)	23.0 [15.0;28.5]	20.5 [14.0;30.5]	23.0 [17.5;37.0]	30.0 [20.0;41.0]	28.0 [23.0;33.0]	26.0 [22.0;40.0]	0.125	**0.032**
CRP (mg/dL)	0.38 [0.15;0.68]	0.73 [0.28;1.71]	0.39 [0.24;1.06]	0.66 [0.17;1.55]	0.54 [0.13;0.99]	0.43 [0.16;0.62]	**0.044**	0.314
Osteocalcin (ng/mL)	7.30 [6.15;11.9]	9.40 [7.85;12.5]	13.4 [10.6;19.0]	6.95 [5.17;9.12]	9.75 [6.65;14.4]	11.8 [7.35;16.9]	**<0.001**	0.635
Vitamin D (nmol/L)	17.5 [10.8;38.2]	43.6 [39.1;56.5]	51.8 [39.7;71.5]	34.0 [15.9;40.6]	30.2 [23.0;43.0]	29.6 [25.1;43.0]	**<0.001**	**<0.001**
HMB (µmol/L)	3.26 [1.54;4.47]	20.0 [6.67;28.2]	5.73 [4.06;34.2]	1.61 [1.19;4.58]	3.73 [1.58;7.51]	2.26 [1.30;5.66]	**0.001**	**0.003**
Leukocytes (1000/µL)	5.2 [4.0;7.0]	4.8 [4.0;5.8]	4.3 [3.9;5.4]	5.0 [3.5;5.7]	4.2 [3.8;5.5]	4.3 [3.3;5.3]	0.055	0.058
Hemoglobin (g/dL)	12.4 [11.1;13.5]	12.0 [10.9;14.0]	12.8 [11.1;13.8]	13.1 [11.3;13.7]	12.9 [11.5;14.1]	12.7 [11.6;13.7]	0.987	0.735
Platelets (1000/µL)	100 [72;132]	75 [68;101]	86 [73;101]	96 [78;112]	105 [84;121]	93 [74;120]	0.556	0.68
INR	1.21 [1.08;1.35]	1.20 [1.17;1.40]	1.19 [1.13;1.31]	1.23 [1.12;1.41]	1.21 [1.11;1.29]	1.22 [1.08;1.26]	0.561	0.914

T0: baseline, T1: 6 wk, T2: 12 wk. Data are median [interquartile range]. LDL: Low-Density Lipoprotein, chol: cholesterol, HDL: High-Density Lipoprotein, APO: apolipoprotein, ALP: Alkaline Phosphatase, GGT: Gamma-GlutamylTransferase, AST: Aspartate Transaminase, ALT: Alanine Transaminase, CRP: C-Reactive Protein, HMB: β-Hydroxy-β-MethylButyrate, INR: International Normalized Ratio. Linear mixed models were used to evaluate the associations of variables with multiple measurements; p*long*: variation over time of each variable for the entire cohort; plong*treatment: treatment-specific longitudinal changes. Bold *p*-values indicate significant differences.

## Data Availability

The data presented in this study are available on request from the corresponding author with prior authorization of our Ethical Committee that can be obtained at https://www.iacs.es/investigacion/comite-de-etica-de-la-investigacion-de-aragon-ceica/ceicaevaluaciones-y-otras-presentaciones (accessed on 22 October 2021).

## Supplementary Materials

The following supporting information can be downloaded at: https://www.mdpi.com/article/10.3390/nu14112344/s1; Table S1: Nutritional composition of each oral supplement; Table S2: Anthropometric characteristics at baseline; Table S3: Laboratory tests at baseline; Table S4: Differences in clinical and body composition characteristics between compliers and dropouts.
