# Supplementary material for: Randomized Clinical Trial: Effects of β-Hydroxy-β-Methylbutyrate (HMB)-Enriched vs. HMB-Free Oral Nutritional Supplementation in Malnourished Cirrhotic Patients
Source: Nutrients. 2022 Jun 3;14(11):2344. doi: 10.3390/nu14112344 (PMC9183090; doi:10.3390/nu14112344)
Supplement: Supplementary file 1 [file nutrients-14-02344-s001.zip › nutrients-1732944-supplementary.pdf]

Supplementary Material

Table S1. Nutritional composition of each oral supplement

	Ensure® Plus Advance (per bottle)	Ensure® Plus High Protein (per bottle)
Energy (Kcal)	330	275
Proteins (g)	20	17.4
Carbohydrates (g)	37	35
Fats (g)	11	7.3
Water (g)	168	177
Vitamin D3 (mcg)	13	2.4
Leucine (g)	1.78	1.54
Isoleucine (g)	0.99	0.814
Valine (g)	1.14	1.012

Table S2. Anthropometric characteristics at baseline

	**HMB Group (n=22)**			**HP Group (n=21)**			**p**
	**Total**	**Men**	**Women**	**Total**	**Men**	**Women**	
**BMI (Kg/m²)**	25.6 ± 4.31	26.4 ± 4.34	24.2 ± 4.18	26.2 ± 3.94	26.3 ± 4.56	26.1 ± 2.94	0.625
**%FM**	18.4 [6.15-25.6]	16.3 ± 9.91	19.6 ± 11.5	18.8 [8.4-26.1]	14.4 ± 8.47	22.4 ± 9.19	0.923
**FFMI (Kg/m²)**	20.8 ± 2.3	21.7 ± 1.78	19.2 ± 2.32	21.4 ± 2.76	22.2 ± 2.76	20.2 ± 2.39	0.403
**SMI (Kg/m²)**	10.8 [9.15- 11.8]	11.5 ± 1.53	8.55 [7.78-9.57]	10.0 [9.1-11.7]	11.5 ± 1.96	8.65 [7.95-9.55]	0.644
**ASMM (Kg)**	24.4 [19.3-28.2]	26.4 ± 3.23	17.1 [15.8-19.5]	22.2 [19.2-27.2]	25.4 ± 5.38	19.3 [17.2-22.4]	0.715
**Biceps skinfold (mm)**	6 [4.5-10]	6.09 ± 2.63	9.12 ± 6.29	8 [6-10]	7.88 ± 4.09	10.0 ± 4.38	0.211
**Triceps skinfold (mm)**	12.9 ± 6.38	10.9 ± 4.27	16 ± 8.18	14.4 ± 6.8	12 ± 6.78	18.2 ± 5.13	0.468
**MUAC (cm)**	26 ± 4.76	27.1 ± 4.64	24.2 ± 4.66	27.7 ± 3.88	27.7 ± 4.43	27.8 ± 3.06	0.207
**MAMC (cm)**	22.2 ± 4.19	23.9 ± 3.89	19.2 ± 2.84	23.2 ± 3.51	24.0 ± 3.89	22.0 ± 2.58	0.381
**Calf circumference (cm)**	35 ± 4.57	36.4 ± 3.21	32.6 ± 5.75	36.2 ± 4.14	36.8 ± 4.75	35.1 ± 2.86	0.385
**Hand grip (Kg)**	28.9 ± 7.48	33.1 ± 5.9	21.5 ± 2.39	31.7 ± 7.93	35.5 ± 6.5	25.5 ± 6.07	0.240

BMI: body mass index, %FM: percentage of fat mass, FFMI: fat-free mass index, SMI: skeletal muscle mass index, ASMM: appendicular skeletal muscle mass, MUAC: mid-upper arm circumference, MAMC: mid-arm muscle-circumference. P: p-value for the comparison between total HMB vs. HP groups.

Table S3. Laboratory tests at baseline

	HMB Group (n=22)	HP Group (n=21)	p
**Ferritin (ng/ml)**	156.0 [60.9-523.0]	97.2 [72.4-662]	0.662
**Transferrin (mg/dl)**	218.0 ± 56.6	217.0 ± 74.1	0.960
**Vitamin B12 (pg/ml)**	488.0 [252-778]	599.0 [389,0-756.0]	0.161
**CRP (mg/dl)**	0.38 [0.15-0.68]	0.66 [0.16-1.55]	0.512
**Urea (mg/dl)**	29.0 [24.5-42.5]	28.0 [25.0-34.0]	0.670
**Creatinine (mg/dl)**	0.73 [0.58-0.91]	0.69 [0.61-0.81]	0.874
**Triglycerides (mg/dl)**	68.0 [51.2-93.2]	68.0 [54.0-106.0]	0.496
**Total cholesterol (mg/dl)**	172.0 ± 58.9	177.0 ± 50.9	0.748
**HDL-cholesterol (mg/dl)**	50.9 ± 19.6	54.6 ± 16.9	0.509
**LDL-cholesterol (mg/dl)**	106.0 ± 44.6	106.0 ± 41.3	0.949
**APO A1 (mg/dl)**	138.0 ± 43.5	143.0 ± 37.3	0.694
**APO B (mg/dl)**	72.3 [51.3-112]	70.3 [63.8-86.1]	0.961
**Lipoprotein (a) (mg/dl)**	9.75 [3.21-19.9]	3.53 [2.07-9.72]	0.155
**Bilirubin (mg/dl)**	1.69 [1.05-2.46]	1.75 [1.31-2.57]	0.662
**Plasma proteins (g/dl)**	7.20 [6.82-7.40]	7.20 [6.60-7.40]	0.644
**Albumin (g/dl)**	3.58 ± 0.57	3.56 ± 0.62	0.913
**Prealbumin (mg/dl)**	11.6 [6.38-15.2]	9.18 [7.62-12.8]	0.846
**ALP (U/L)**	104.0 [93.8-140.0]	131.0 [112.0-158.0]	0.138
**GGT (U/L)**	64.5 [40.2-100.0]	98.0 [61.0-126.0]	0.194
**AST (U/L)**	40.5 [30.8-56.5]	43.0 [32.0-88.0]	0.248
**ALT (U/L)**	23.0 [15.0-28.5]	30.0 [21.0-41.0]	0.018
**Ammonia (µM)**	56.0 [40.0-83.0]	55.0 [40.0-76.0)	0.923
**Vitamin D (nmol/L)**	17.5 [10.8-38.2]	34.1 [16.3-42.6)	0.195
**BAP (U/L)**	24 [19.5-29.9]	26.8 [23.6-29.1]	0.325
**Osteocalcin (ng/ml)**	7.3 [6.15-11.9]	7.0 [5.6-9.5]	0.415
**PICP (µg/l)**	123.0 [97.7-175.0]	118.0 [88.4-145.0]	0.529
**CTX (pg/ml)**	443.0 [285.0-603.0]	289.0 [250.0-424.0]	0.033
**HMB (µmol/l)**	3.26 [1.54-4.47]	1.5 [1.19-3.17]	0.099

CRP: C-Reactive Protein, HDL: High-Density Lipoprotein, LDL: Low-Density Lipoprotein, APO A1: Apolipoprotein A1, APO B: Apolipoprotein B, ALP: Alkaline Phosphatase, GGT: Gamma-GlutamylTransferase, AST: Aspartate Transaminase, ALT: Alanine Transaminase, BAP: Bone Alkaline Phosphatase, PICP: Procollagen type I Carboxy-terminal Propeptide, CTX: C-Terminal telopeptide, HMB: ß-Hydroxy-ß-MethylButyrate

Table S4. Differences in clinical and body composition characteristics between compliers and dropouts

	Compliers (n=34)	Dropouts (n=9)	p
**Age (Years)**	61.1 (9.09)	60.1 (8.31)	0.756
**Men (n%)**	13 (38.2%)	3 (33.3%)	1.000
**Cirrhosis Etiology n (%)**			
**Alcohol**	21 (61.8%)	7 (77.8%)	0.624
**HCV**	4 (11.8%)	1 (11.1%)	
**Autoimmune**	3 (8.82%)	1 (11.1%)	
**NAFLD**	3 (8.82%)	0 (0.00%)	
**HBV+NAFLD**	1 (2.94%)	0 (0.00%)	
**PBC**	1 (2.94%)	0 (0.00%)	
**Hemochromatosis**	3 (8.82%)	1 (11.1%)	
**Ascites**	18 (52.9%)	3 (33.3%)	0.457
**Child-Pugh**	7.00 [6.00;8.75]	6.00 [6.00;8.00]	0.581
**MELD**	11.5 [9.25;15.8]	15.0 [7.00;18.0]	0.787
**SGA**	9.75 [3.21-19.9]	3.53 [2.07-9.72]	0.155
**Class B**	1.69 [1.05-2.46]	1.75 [1.31-2.57]	0.662
**Class C**	7.20 [6.82-7.40]	7.20 [6.60-7.40]	0.644
**BMI**	25.7 (4.32)	26.5 (3.24)	0.557
**FMI**	5.20 [1.03;7.27]	4.60 [3.30;6.40]	0.846
**FFMI**	21.0 (2.51)	21.5 (2.70)	0.589

Data are number (%), median [IQR], or mean ±SD. p: p-value for the difference. HCV: Hepatitis C Virus, NAFLD: Non-Alcoholic Fatty Liver Disease, HBV: Hepatitis B Virus, PBC: Primary Biliary Cholangitis, MELD: Model for End Stage Liver Disease, SGA: Subjective Global Assessment, BMI: body mass index, FMI: fat mass index, FFMI: fat-free mass index
